# Immune Profiling in Gastric Cancer Reveals the Dynamic Landscape of Immune Signature Underlying Tumor Progression

**DOI:** 10.3389/fimmu.2022.935552

**Published:** 2022-07-08

**Authors:** Yuhan Wei, Jianwei Zhang, Xueke Fan, Zhi Zheng, Xiaoyue Jiang, Dexi Chen, Yuting Lu, Yingrui Li, Miao Wang, Min Hu, Qi Du, Liuting Yang, Hongzhong Li, Yi Xiao, Yongfu Li, Jiangtao Jin, Deying Wang, Xiangliang Yuan, Qin Li

**Affiliations:** ^1^ Department of Oncology, Beijing Friendship Hospital, The Second Clinical Medical College of Capital Medical University, Beijing, China; ^2^ Department of Pancreatic and Gastric Surgery, National Cancer Center/Cancer Hospital, Chinese Academy of Medical Sciences and Peking Union Medical College, Beijing, China; ^3^ Gastroenterology Department, Jincheng People’s Hospital, Jincheng, China; ^4^ Department of General Surgery, Beijing Friendship Hospital, Capital Medical University, Beijing, China; ^5^ Beijing Institute of Hepatology, Beijing You An Hospital, Capital Medical University, Beijing, China; ^6^ Biochemistry and Molecular Biology, Basic Medical College, Shanxi Medical University, Taiyuan, China; ^7^ Chongqing Key Laboratory of Molecular Oncology and Epigenetics, First Affiliated Hospital of Chongqing Medical University, Chongqing, China; ^8^ Department of Molecular and Cellular Oncology, The University of Texas MD Anderson Cancer Center, Houston, TX, United States; ^9^ Department of Oncology, The Second Affiliated Hospital of Hainan Medical University, Haikou, China; ^10^ Department of Intervention Therapy, Zezhou People’s Hospital, Jincheng, China; ^11^ Department of Technology Innovation, China National Nuclear Corporation (CNNC) Hexin Information Technology (Beijing) Co., Ltd., Beijing, China; ^12^ Department of Laboratory Medicine, Ruijin Hospital, Shanghai Jiao Tong University School of Medicine, Shanghai, China

**Keywords:** immune profiling, gastric cancer, PD-1, biomarker, tumor immune microenvironment

## Abstract

The profiling of the tumor immune microenvironment (TIME) is critical for guiding immunotherapy strategies. However, how the composition of the immune landscape affects the tumor progression of gastric cancer (GC) is ill-defined. Here, we used mass cytometry to perform simultaneous in-depth immune profiling of the tumor, adjacent tissues, and blood cells from GC patients and revealed a unique GC tumor-immune signature, where CD8^+^ T cells were present at a lower frequency in tumor tissues compared to adjacent tissues, whereas regulatory T cells and tumor-associated macrophages (TAMs) were significantly increased, indicating strong suppressive TIME in GC. Incorporated with oncogenic genomic traits, we found that the unique immunophenotype was interactively shaped by a specific GC gene signature across tumor progression. Earlier-stage GC lesions with IFN signaling enrichment harbored significantly altered T-cell compartments while advanced GC featured by metabolism signaling activation was accumulated by TAMs. Interestingly, PD-1 expression on CD8^+^ T cells was relatively higher in earlier-stage GC patients, indicating that these patients may derive more benefits from PD-1 inhibitors. The dynamic properties of diverse immune cell types revealed by our study provide new dimensions to the immune landscape of GC and facilitate the development of novel immunotherapy strategies for GC patients.

## Introduction

Immunotherapy has become highly successful against cancers by triggering or restoring the cytotoxic potential of the human immune system (1–3). Among cancer immunotherapies, immune checkpoint blockade (ICB) which targets cytotoxic T lymphocyte antigen 4 (CTLA-4) or the programmed cell death 1 (PD1)–programmed cell death ligand 1 (PD­L1) axis has been approved for the treatment of several different cancer types (4–7). Despite the great achievements ICB has made, clinical positive responses have only been observed in a small fraction of patients and most patients including patients with gastric cancer (GC) still do not obtain a meaningful response to it (2, 3, 8). GC is the fifth most common cancer and the fourth leading cause of cancer-related deaths worldwide (9, 10), and the progression of GC was demonstrated to be strongly correlated with the immune response (11, 12). Therefore, the adoption of more extensive immunogenetic profiling of tumor-infiltrating immune cells in GC is expected to pave the way toward understanding the integrated tumoral immune system as well as help discover precision immunotherapy to fight against GC.

The tumor immune microenvironment (TIME) is a heterogeneous and complex system with continuous changes in the progression of tumor initiation, growth, and dissemination. Cells of the adaptive and innate immune systems infiltrate TIME and form an ecosystem that modulates all aspects of tumor development. CD4+ helper T cells and cytotoxic CD8+ T cells can prevent tumor growth by targeting antigenic tumor cells, and high numbers of activated CD8+ T cells are associated with a good prognosis in GC and various cancers (13, 14). Meanwhile, tumor-infiltrating T lymphocytes (TILs) also include a population of regulatory T (Treg) cells, a subset of CD4+ T cells, which accumulate in TIME and suppress tumor-specific T-cell responses (15, 16). Tumor-associated macrophages (TAMs) and dendritic cells (DCs), as the main components of antigen-presenting cells, play an indispensable role in the adaptive immune response by capturing and presenting tumor antigens to CD4+ and CD8+ T cells. Besides, TAMs are the major constituent of immune cells in the TIME that can either block or facilitate tumor growth in many cancers (17). Growing evidence has shown a strong association between TAM density and poor prognosis in GC and other solid cancers (18–22). Recently, B cells were identified to play an important role in antitumor immune response in TIME (23–25). In addition, innate immune cells such as natural killer (NK) cells are proved to be important as well (26). An in-depth analysis of complexity within the TIME is likely to reveal the mechanism of tumor immune evasion that will prove fruitful in understanding the tumor progression and will benefit the search for novel targets for therapeutic modulation. However, the thorough composition of GC immune landscape and its effects on tumor progression remain exclusive. Thus, it is important to characterize the baseline GC immune milieu to clarify the composition and property of tumor-infiltrating immune cells in comparison with ones in other immune-relevant anatomical compartments.

In the study, using mass cytometry by time of flight (CyTOF) combined with genomic bioinformatic analysis, we present a paired single-cell analysis of the immune landscape in tumor samples and matched adjacent tissues as well as peripheral blood cells from GC patients. We identify the characterized immune landscape that is unique to GC tumor lesions. We further identified the specific intrinsic tumor features that significantly associate with their immune infiltrate. Our findings provide a landscape of the interactions between tumor and immune cells across gastric cancer. Given the unmet need in developing TIME-targeted therapies for gastric cancer, this comprehensive analysis of the immune landscape in GC offers insights into the interpretation of the response of gastric cancer to ICB and possible personalized strategies to overcome tumor-supporting TIME properties and thereby guide the development of novel drug combination strategies.

## Results

### Single-cell Mapping of Immune Landscape in Human Gastric Cancer by CyTOF

**Figure 1:**
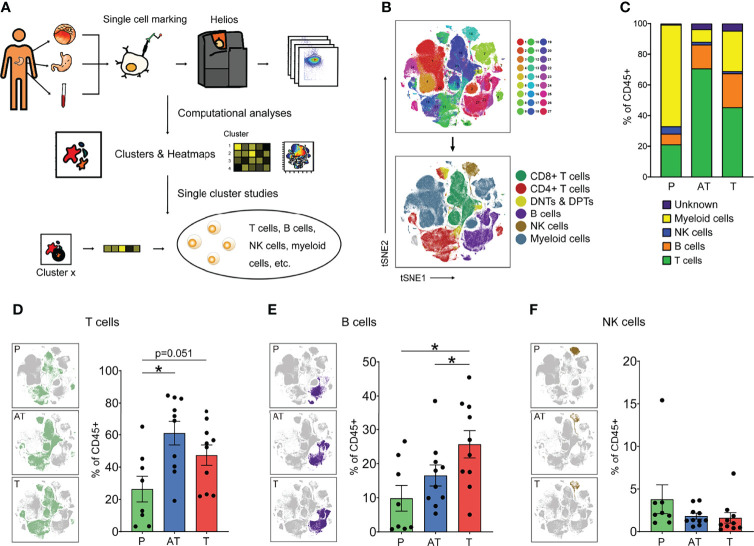
The immune landscape of human gastric cancer (GC) by CyTOF. **(A)** Experimental approach of the study. **(B)** tSNE maps displaying immune cells of GC patients colored by 27 Phenograph clusters (top) and the main cell populations by manual identification of Phenograph clustering (below). **(C)** Average frequencies of major immune lineages for each type of sample (peripheral blood mononuclear cells [PBMCs, P], n = 8; adjacent tissues [AT] and tumor tissues [T], n = 10) across GC patients. **(D–F)** Distribution of the T **(D, B, E)**, and NK **(F)** cells in different types of samples (left) and frequency of that for each patient (right) based on manual identification of Phenograph clusters. Bar plots show mean ± SEM; *p < 0.05 by paired t-test. P, peripheral blood mononuclear cells (PBMCs); AT, adjacent tissues; T, tumor tissues.

To generate in-deep immunophenotyping of the immune cell states in human gastric cancer, we performed a large-scale mass cytometry analysis of 20,000 CD45+ immune cells for each sample collected from 10 primary tumor samples from patients undergoing surgery with all grades of GC (**Figure 1A**). Clinical information of all GC patients that were representative of the gastric adenocarcinoma distribution across age, gender, and predominant histological subtype was summarized in **Supplementary Table S2**. To explore tumor-specific immune changes from the gastric immune microenvironment, we sought to simultaneously map the immune compartment of the tumor lesion, adjacent tissue, and peripheral blood of GC (**Figure 1A**). To this end, we designed a comprehensive CyTOF panel together, measuring 37 parameters at the single-cell level that allowed simultaneous analysis of cells from all three sample types, to deeply interrogate the lymphoid compartment and capture the entire spectrum of myeloid populations, together with lineage-identifying and functional markers to map the cellular frequencies of major immune cell populations and their functional status (**Supplementary Figure S1A**).

This strategy allowed us to visualize high-dimensional data in two dimensions and systematically identify the main immune cell types in both lymphocytes and myeloid cells across the three tissues of all patients (**Figure 1B** and **Supplementary Figures S1B–D**). Totally, the immune cell compartment in GC tissues comprised all major immune lineages. Interestingly, we observed the diverse situations in tumor lesions of GC, indicated by lower relative frequencies of T cells (47.3%) and higher frequencies of myeloid cells (22.6%), compared with 61.0% and 15.1% in adjacent tissues (**Figure 1C** and **Supplementary Figures S1E, F**). Our findings highlight that tissue of residence is a significant determinant of immune phenotype, showing that GC shapes TIME with a distinct immune cell composition: myeloid cells dominate the TIME in tumor lesions whereas lymphocytes dominate in adjacent tissues. A close analysis of the immune compartment of TIME allowed us to distinguish the specific immune change in GC.

### Unique Characterization in Lymphocyte Composition of GC

As lymphocytes represent the most abundant cell subsets across GC tumor lesions and adjacent tissues and are considered the most clinically impactful for antitumor immunity and immunotherapy, we focused subsequent in-depth analyses on these major cell types (**Figure 1C**). Upon t-SNE dimensionality reduction in conjunction with Phenograph clustering, we identified three major compositions of lymphocytes, namely, T cells (CD3+), B cells (CD19+), and NK cells (CD3-CD56+) (**Figures 1D–F**). Across all samples, T and B lymphocytes were present at a higher frequency in the GC tumor microenvironment and adjacent tissue microenvironment compared to blood (**Figures 1D, E**), whereas the frequency of NK cells was slightly reduced across all GC patients examined (**Figure 1F**).

Notably, it showed the tendency among the GC patients examined that B lymphocytes were present at a higher frequency in the GC tumor microenvironment compared to the adjacent tissues (**Figure 1E**). Tumor-infiltrating B cells are heterogeneous, and their roles in tumor immunity remain elusive. Specifically, in our study, total B cells were significantly increased in GC tissues compared to adjacent tissues and peripheral blood; however, we found that the CXCR5+ B cell (cluster 10) is decreased in GC tissues and other clusters of B cells were significantly increased (**Supplementary Figure S1H, I**).

### T-cell Status Reveals Various Phenotypes Associated with GC Immunosuppression

**Figure 2:**
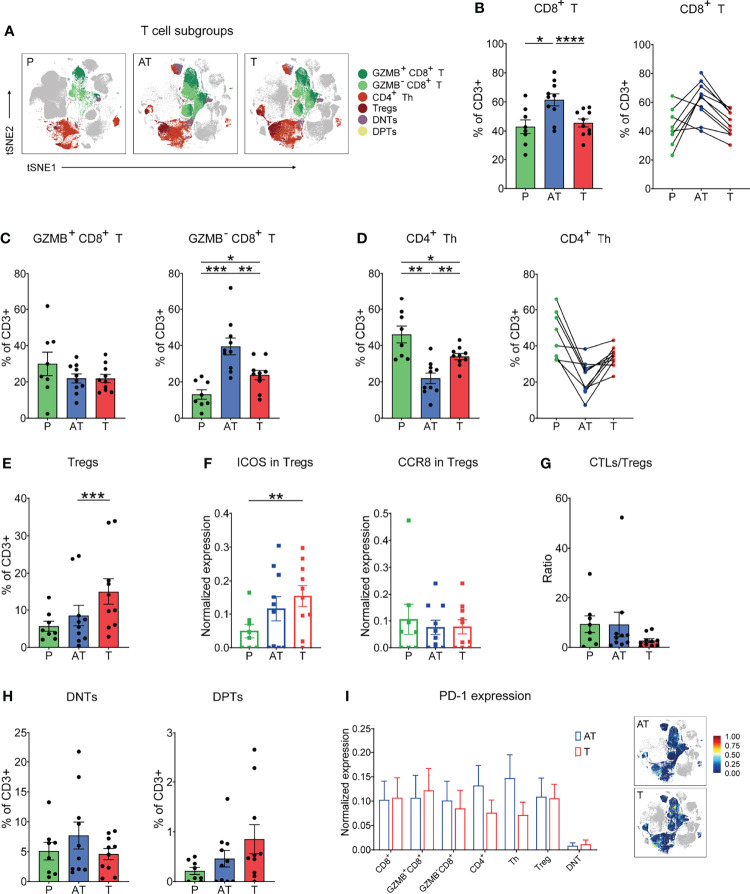
In-depth characterization of the amount and function of T-cell (CD3^+^) compartments in the three types of samples (% of CD3^+^ T cells). **(A)** tSNE plots showing the distribution of the T-cell subgroups in different types of GC samples. **(B)** Frequency (left) and individual characterization (right) of CD8^+^ T cells for each patient based on summation of Phenograph clusters. **(C)** Frequency of GZMB^+^CD8^+^ (left) and GZMB^-^CD8^+^ (right) T-cell clusters for each patient based on summation of Phenograph clusters. **(D)** Frequency (left) and individual characterization (right) of CD4^+^ helper T (Th) cells for each patient based on summation of Phenograph clusters. **(E)** Frequency of Tregs for each patient based on summation of Phenograph clusters. **(F)** Expression level of ICOS and CCR8 in Tregs for each patient based on summation of Phenograph clusters. **(G)** Cytotoxic T lymphocyte (CTLs)/Treg ratio for each patient based on summation of Phenograph clusters. **(H)** Frequency of CD4^-^CD8^-^ double-negative T (DNT) and CD4^+^CD8^+^ double-positive T (DPT) cells for each patient based on summation of Phenograph clusters. **(I)** Expression level of PD-1 in different T-cell subgroups (left) and tSNE maps of relative expression of the PD-1 for T cell subgroups in GC samples of adjacent tissues and tumor tissues. Bar plots show mean ± SEM; *p < 0.05, **p < 0.01, ***p < 0.001, and ****p < 0.0001 by paired t-test.

T cells were the main immune cell population in the GC TIME. Here, upon t-SNE dimensionality reduction in conjunction with Phenograph clustering, we in-depth identified five major compositions of T cells including CD8+ cytolytic T cells (CD3+CD8+, CTL), CD4+ T help cells (CD3+CD4+CD25-, CD4+ Th), regulatory T cells (CD3+CD4+CD25+Foxp3+, Tregs), double-negative T cells (CD3+CD4-CD8-, DNT), and double-positive T cells (CD3+CD4+CD8+, DPT) (**Figure 2A**). Interestingly, despite infiltration of more CD8+ T cells in tissues than in PBMCs, it seemed that CD8+ T cells were limited in adjacent tissues rather than GC tissues (**Figure 2B**, and **Supplementary Figure S2A**). Besides, it was proved that Granzyme B (GZMB)-CD8+ T cells significantly increase in tumor tissues compared with PBMCs while GZMB+CD8+ T cells showed no significant difference, and the expression of GZMB on CD8+ T cells was observed significantly lower in tumor tissues than in peripheral blood, which may indicate both tumor immune resistance-induced spatial limitation and dysfunction of CD8+ T cells (**Figure 2C**, and **Supplementary Figures S2B, C**). However, the reduction of GZMB-CD8+ T cells was more pronounced compared to GZMB+CD8+ T cells in cancerous tissues (**Figure 2C**, and **Supplementary Figure S2B**). In contrast to CD8+ T cells, CD4+ Th cells were of a lower frequency in the GC lesions and adjacent tissues compared to blood (**Figure 2D**, and **Supplementary Figure S2D**). Specifically, Tregs were major components of CD4+ T cells and significantly accumulated in the tumor lesion across all GC patients (**Figure 2E**, and **Supplementary Figure S2D**). Moreover, Tregs present in GC and adjacent tissues expressed high ICOS compared to blood, with no change in CCR8 expression (**Figure 2F**). Remarkably, the CTL/Treg ratio was significantly lower in the tumor lesions of GC than in adjacent tissues and blood (**Figure 2G**). This characterization of T subsets strongly indicates the suppressive microenvironment in TIME of GC. Immunohistochemical results showed less infiltration of T cells in tumor tissues than that in adjacent tissues for GC patients (**Supplementary Figure S2E**).

Among the T-cell components, two specific forms were identified, double-negative CD4-CD8- (DNTs) and double-positive CD4+CD8+ population (DPTs), which were observed across all GC samples, reaching up to 5%–10% and 0.5%–2.5% of the T-cell compartment in GC tissues, respectively (**Figure 2H**). Despite that the origin of DNTs has not been fully unveiled, our previous report showed that DNTs have emerged as functional immune cells in the field of antitumor therapy due to their high cytotoxicity in multiple tumor cells (27). In this study, we found that the frequency of DNTs in GC tissues displayed a lower tendency compared to that of adjacent tissues (**Figure 2H**). The exact function of DNTs and DPTs for GC progression or potential immunotherapeutic application still needs to be identified.

In view of the promising efficacy of anti-PD-1 therapy in several cancer patients, we examined the expression of the checkpoint molecule PD-1 on all distinct T subsets and found that the PD-1 expression on T-cell subsets, particularly CD8+ T cells, between cancer tissues and adjacent tissues was basically comparable (**Figure 2I**).

### The GC TIME Harbors a Heterogeneous Myeloid Cell Population

**Figure 3:**
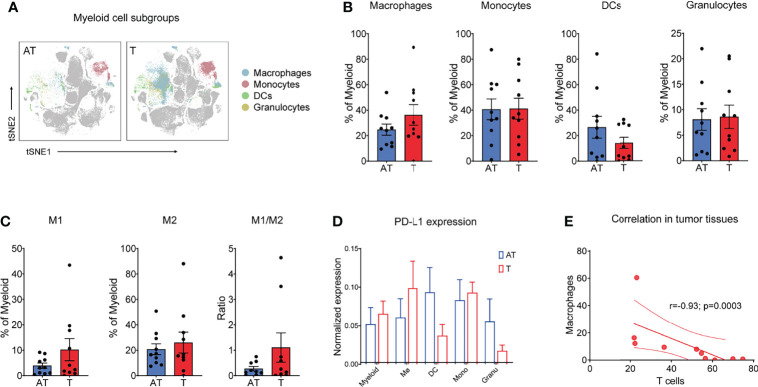
Myeloid cells show a distinct phenotype in a GC-immune microenvironment. **(A)** tSNE plots showing the distribution/localization of the myeloid cell subgroups in adjacent tissues and tumor tissues. **(B)** Frequency of macrophages, monocytes, dendric cells (DCs), and granulocytes for each patient based on summation of Phenograph clusters. **(C)** Frequency of M1 and M2 types of macrophages and M1/M2 ratio for each patient based on summation of Phenograph clusters. **(D)** Expression level of PD-L1 on different T-cell subgroups (left) and tSNE maps of relative expression of the PD-L1 for myeloid cell subgroups in GC samples of adjacent tissues and tumor tissues. **(E)** Correlation between T cells and macrophages in all tumor tissues. Spearman’s correlation r and p values are shown. Bar plots show mean ± SEM. .

Although myeloid cells are key components of the tumor microenvironment, their heterogeneity and impact on GC progression remain insufficiently characterized. Phenograph clustering of all tissues in GC patients confirmed the diversity of myeloid cells in GC tumors, and macrophages, monocytes, DC subsets, and neutrophil subsets were identified based on the expression of protein markers, including CD11b, CD68, CD163, CD206, CD16, CD14, CD11c, CD141, PPARγ, and HLA-DR, among others. Paired mass cytometry analysis revealed a distinct composition and phenotype of myeloid populations between tumor and adjacent tissue samples (**Figure 3A**). Specifically, macrophages displayed an increased tendency in the tumor lesion across all GC patients, while dendritic cells (DCs) reduced in cancer tissues compared with adjacent tissues (**Figure 3B** and **Supplementary Figure 3A**). Granulocytes and monocytes accounted for approximately the same proportion between tumor and adjacent tissues (**Figure 3B** and **Supplementary Figure S3A**).

Notably, TAMs and monocytes comprised 77.4% of myeloid cells in the tumor lesions, which was higher than those in non-tumor adjacent tissues (**Figure 3B**). Using paired CyTOF analyses, we found that tumor macrophages exhibited a distinct signature in GC compared to their adjacent counterparts across all patients (**Figure 3B**). Importantly, while M2-type macrophages dominated gastric cancer that was in line with previous studies (21, 22), the increased proportion of macrophages in tumor sites was mainly due to the enrichment of M1-type macrophages (**Figure 3C** and **Supplementary Figure S3B**). In addition, the M1/M2 rate tended to be higher in cancer tissues in comparison to that in adjacent tissues, although without statistical significance (**Figure 3C**). Tumor macrophages expressed higher levels of CD68 and PPARγ and lower levels of HLA-DR compared to components that resided in adjacent tissues (**Supplementary Figure S3C**). Using paired CyTOF analyses, we identified the presence of three DC subsets (cDC1, cDC2, and pDCs) at the tumor site across GC patients. DCs, as professional antigen presentation cells (APC), excel at antigen presentation and play a critical role in the induction of antitumor T-cell immunity. Strikingly, CD141+ DCs, cross-presenting cells that preferentially prime CD8+ T cells, were significantly reduced in tumor lesions compared to adjacent tissues (**Supplementary Figure S3D**), which consistent with the desert status of CD8+ T cells in GC tumor tissues (**Figures 2B, C**). These findings indicated the deficiency of specific antitumor immunity in GC tumor sites. Tumor-infiltrating myeloid cells can express PD-L1, which is a key regulator of T-cell immunity in cancer (28, 29). We detected PD-L1 expression on distinct myeloid subsets and found that macrophages highly express PD-L1, while the expression of PD-L1 in DCs was relatively lower in cancer tissues (**Figure 3D**).

To quantify relationships between immune cell components presented in the TIME of GC, we calculated the frequencies for major immune cell phenotypes across all GC patients. Interestingly, we found that the main pro-tumor TAM clusters displayed a mutually exclusive relationship with CD8+ T cells, whereas this negative correlation only happened in the tumor lesions (r = -0.93, P = 0.0003) (**Figure 3E**), not in adjacent tissue (**Supplementary Figure S3E**). These differences of tumor lesions compared to adjacent tissues likely work in concert to exhibit an immunosuppressive microenvironment.

### Dynamic Signature of the Immune Landscape Underlying GC Tumor Progress and Clinical Outcomes

**Figure 4:**
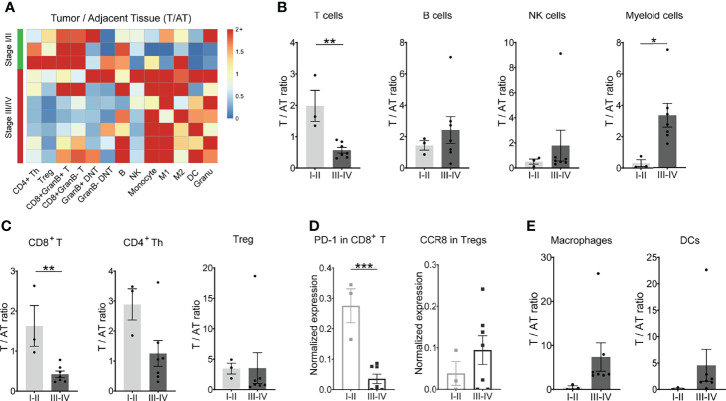
Dynamic signature of the immune landscape underlying GC tumor progression. **(A)** Heatmap showing the relative differences in main immune cell lineages between tumor tissues and adjacent tissues across 10 patients, grouped by stage. Ratio ≥2 was shown as 2+. **(B)** Ratio of T, B, NK, and myeloid cells between tumor tissues and adjacent tissues (T/AT) for each patient by stage. **(C)** Ratio of CD8^+^ T cells, CD4^+^ Th cells, and Treg cells between tumor tissues and adjacent tissues (T/AT) for each patient by stage. **(D)** Expression level of PD-1 in CD8^+^ T cells and CCR8 in Tregs for tumor tissue of each patient by stage. **(E)** Ratio of macrophages and DCs between tumor tissues and adjacent tissues (T/AT) for each patient by stage. Bar plots show mean ± SEM; ***p < 0.001 by paired t-test.

To obtain an overall profile of the tumor-immune systems in response to gastric cancer, we analyzed the immune landscape of diverse tumor-infiltrating immune cells across tumor progression based on TNM stage (T—tumor; N—node; M—metastasis) classification. We examined whether the immune contexture of the tumor lesions significantly differed between earlier-stage (stage I/II) and later-stage (stage III/IV) tumors. Intriguingly, the distribution of major immune cell subsets including T-cell subsets, B cells, and NK cells as well as macrophages and DCs showed different trends in frequency across different stages (**Figure 4A** and **Supplementary Figure S4A**). Tumor lesions with earlier-stage gastric cancer harbored significantly altered T-cell compartments in the tumor immune microenvironment compared with adjacent tissues, which was characterized by higher infiltrations of CD8+ T cells and a predominant phenotype of CD4+ T cells (**Figures 4A–C**; **Supplementary Figures S4B, S4C**. However, the later-stage GC lesions were characterized by tumor-infiltrating myeloid cell subsets, which likely compromise antitumor T-cell immunity (**Figures 4A, B**).

Besides fewer infiltrations of CD8+ T cells in the TME, the expression level of PD-1 molecules on CD8+ T cells was relatively lower in the later-stage GC patients in comparison with early-stage GC patients (**Figure 4D**). CD4+ Th cells, Treg, and DNT did not display a significant difference across the tumor progress of GC (**Figure 4C**; **Supplementary Figure S4C**). However, chemokine receptor CCR8 was highly expressed by Treg cells in later-stage GC patients in comparison to that in earlier-stage GC patients (**Figure 4D**), indicating a strong chemotaxis of tumor cells or microenvironment components on Tregs in GC patients with advanced stage. For the mononuclear phagocytes, there was no significant difference for both macrophages and DCs (**Figure 4E**; **Supplementary Figure S4D**).

To obtain a global understanding of the relationships between all immune subsets and characteristics of the tumors, we compared the immune feature in GC patients with or without recurrence and metastasis based on 2 years of following up. Similarly, recurrence patients exhibited the comparable immune signatures with later-stage GC patients, as indicated with no dramatic change regarding the frequency of total T, B, NK, and myeloid populations (**Supplementary Figures S4E, F**).

### Genomic Traits of GC Modulate Tumor Immunophenotypes and Response to Checkpoint Blockade

**Figure 5:**
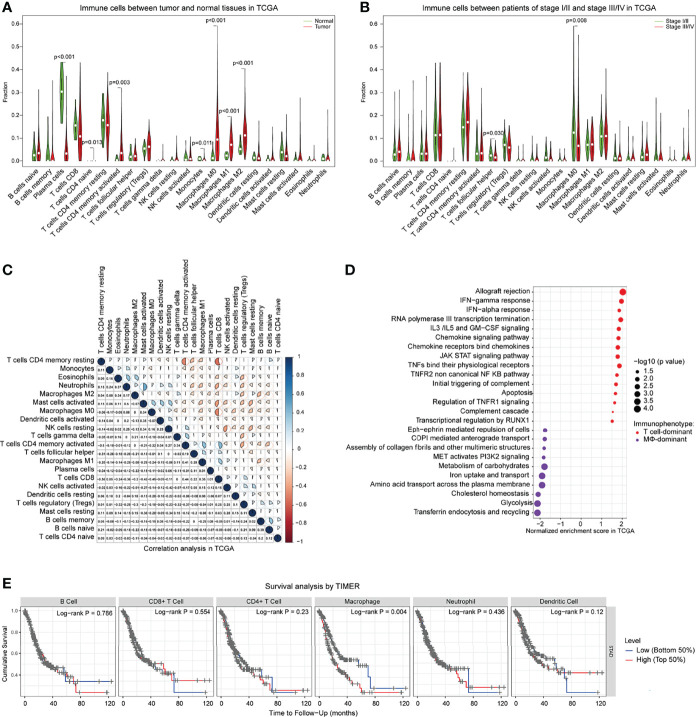
Genomic traits of GC-determined immunophenotypes and clinical information in TCGA. **(A)** Comparisons of the 22 immune cells defined by CIBERSORT between tumor and adjacent normal tissues of GC patients for all eligible samples in TCGA. **(B)** Comparisons of the 22 immune cells between GC patients with stage I/II and stage III/IV in TCGA. **(C)** Correlation of various immune cells defined by CIBERSORT in TCGA. **(D)** Selected pathways related to the T cell-dominant immunophenotype (T cells/macrophages ≥1) and macrophage-dominant immunophenotype (T cells/macrophages <1) by GSEA. Pathways for which |NES|>1, p < 0.05, are chosen to be shown. The position of each circle represented the normalized enrichment score of immunophenotype in which the upregulated pathway is detected in GC patients. The size of the circles represents -log10 (*P*-value). **(E)** Kaplan–Meier curve showing the overall survival based on the level of various immune cells by TIMER. GSEA, gene set enrichment analysis; NES, normalized enrichment score; TIMER, tumor immune estimation resource.

To identify genomic and transcriptomic traits associated with these immunophenotypes in GC, we performed an extensive immunogenomic analysis of GC tumors by utilizing publicly available cancer genomics data compiled by TCGA. To this end, we first computed the relative abundance of an array of 22 main immune-cell populations in GC tumor tissues and adjacent normal tissues. In concordance with CyTOF data, we observed that among 22 inferred immune cell types by CIBERSORT, the higher cellular fractions of CD4+ T cells, especially the memory CD4+ T cells, and macrophages (M0, M1, M2) eventually increased in tumor sites compared to normal tissues (**Figure 5A** and **Supplementary Figure S5A**). This pattern of immune signature was validated by other GC cohorts (GEO) and reached a similar pattern (**Supplementary Figure S5B**). In agreement with CyTOF data, a higher fraction of macrophages was associated with a more aggressive phenotype (**Figure 5B**, Supplementary S5C). This analysis revealed that the relative abundance of many immune cell populations is correlated (**Figure 5C** and **Supplementary S5D**), suggesting at least some degree of co-infiltration of the GC tumor.

Incorporated with CyTOF data, these analyses of genomic traits suggested that tumor cells might have reprogrammed the TIME to facilitate GC progression. We reasoned that the component of the infiltration of the immune cells was the main responsible for the selective pressure applied on GC tumors, resulting in the positive selection of specific features that support the escape of immunosurveillance. Based on TCGA data analysis, we clustered tumors with similar infiltration patterns and defined two main immunophenotype groups: T-cell dominant and macrophage dominant (**Figure 5D**). Next, we intended to identify the genomic features of GC tumors associated with these two immunophenotypes. All subsequent analyses of the gene set enrichment analysis (GSEA) pathway enrichment was stratified across both immunophenotypes. Strikingly, several cancer hallmark signaling pathways were nearly universally upregulated in T-cell-dominated phenotypes (**Figure 5D**), including a marked enrichment of IFN-α and IFN-γ responses, chemokine signaling pathway, JAK-STAT signaling pathway, TNF pathway, complement system, inflammatory response, and apoptosis. On the other hand, enrichment of PI3K2 signaling, transferrin endocytosis and recycling, glycolysis, and iron uptake/transport was observed in the macrophage-dominated immunophenotypes (**Figure 5D**), in agreement with a previous study showing the relationship of iron metabolism with macrophage function (30). Particularly, the cholesterol homeostasis was more frequent in macrophage-dominant immunophenotypes, in line with our recent report that lipid metabolism of tumors regulates macrophage polarization (21). Notably, the GSEA pathway enrichment assay across distinct tumor progression displayed similar patterns with macrophage-dominant immunophenotypes with enriched metabolism signaling pathways (**Supplementary Figure S5E**). Finally, we also observed that tumors of more macrophages which accumulated at diagnosis were significantly associated with poor prognosis in GC patients (**Figure 5E**). These observations suggest that tumors preferentially progress in the presence of a high macrophage infiltrate. In contrast, tumors with a favorably T-cell immunophenotype would be partially kept in check by the immune system and progress less frequently to advanced stages.

## Discussion

Therapy resistance and the lack of rational therapeutic targets represent the major obstacles in improving the survival of patients with GC (31). It is widely appreciated that immunosurveillance is a fundamental property of cancer contributing to the development of distant metastases and therapeutic failure (32). Therefore, deeper dissection of TIME is critical for understanding the mechanisms driving the poor prognosis of GC and for overcoming therapeutic resistance. In this study, we dissected, at single-cell resolution, the cellular and transcriptomic TIME of GC tumors using the cutting-edge CyTOF approaches, in combination with integrative computational bioinformatic analyses.

Previous studies have drawn the immune atlas of various tumors such as lung cancer and kidney cancer by mass cytometry (33–35), guiding the immunotherapy of related cancers. In this study, we conducted paired single-cell analyses of the immune cells to deeply interrogate the immune landscape of TIME in gastric cancer. We distinguished the immune changes driven by the tumor lesion from those driven by the gastric tissue, emphasizing the relevance of this type of analysis for the study of the unique tumor microenvironment of GC. Strikingly, GC tumor lesions had strongly reduced CD8+ T effector cells and a significant expansion of Treg at the tumor site compared to adjacent tissues. This reduced CD8+ T effector cell and increased Treg may be useful as a refined biomarker of disease course or response to treatment (15, 16). We further examined the expression of PD-1 on all different T subgroups and found no significant difference in PD-1 expression between cancer tissues and adjacent tissues. These findings may partially explain the failure of anti-PD1 monotherapy in GC patients and further indicated that the key feature of CD8+ T cells in GC is the low frequency, not the defect of function by PD-1, meaning that increasing the recruitment of CD8+ T-cell infiltration in tumor lesions is likely more critical to boosting the antitumor immunity rather than antagonizing PD-1 alone for GC patients. The distinct TIL tumor signature was accompanied by significant alterations of tumor B cells, as they were strongly increased in tumors. Currently, the research on antitumor immunity has been mainly focused on T cells, while the important role of B cells has been overlooked. Previously, we reported that a subset of CD19+CD24hiCD38hi B cells (Breg) increased in GC and played an immunosuppressive role in gastric cancer by inhibiting T-cell cytokines as well as conversion to Tregs (36). Recently, several studies have revealed the positive effect of B cells in the tumor microenvironment, showing that the presence of B cells and tertiary lymphoid structures in tumors was associated with favorable outcomes for immunotherapy (23–25, 37, 38). The concise subsets and function of B cells in GC progression and the correlation with immunotherapy need to be clarified in the future. Notably, we also identified distinct deficiency in antigen-presenting cell subsets that resided in GC tumor lesions across all GC patients.

A key finding of this study is the dynamic major changes in two major populations of T cells and TAMs, which were dictated by the stage of GC. Interestingly, T lymphocytes are abundant in tumor tissues compared to adjacent tissues in the earlier-stage GC, whereas immunosuppressive TAMs dominate the immune landscape of later-stage GC. These results showed that the immune landscape was shaped by tumor progression of gastric cancer and suggested that for later-stage GC patients, overcoming the immunosuppression of TAMs might be a promising therapeutic strategy as the remarkable accumulation of TAMs plays a major role in limiting effective antitumor T-cell immunity in later-stage GC lesions (17, 39, 40). The current armamentarium to treat advanced GC patients is highly deficient. The extensive finding revealed by our study will tailor precision immunological therapies for patients with advanced GC. Moreover, in early-stage GC tumor lesions, a relatively higher amount of PD-1 molecules on CD8+ T cells was found, which indicated that current PD-1 inhibitor ICB might benefit early-stage GC patients. Recently, in a phase 3 randomized clinical trial, ICB immunotherapy showed a favorable benefit-to-risk profile in patients with advanced G/GEJ cancer with a PD-L1-combined positive score (CPS) of 1 or greater in the first-line setting (41). This treatment strategy of PD-1 inhibitor ICB for early-stage cancer patients was successfully incorporated in non-small cell lung cancer (NSCLC) (42), and our study warranted the potential of this strategy for early-stage GC patients.

Additionally, our study further comprehensively characterizes certain genomic and transcriptomic features of GC tumors underlying the tumor-specific immune landscape of TIME across the tumor progression of GC patients. We identified the specific tumor features of GC that are positively selected due to their interaction with the immune infiltrate. In this regard, GCs with a highly T cell-dominant immunophenotype would be at their early stages and equipped with IFN and inflammatory signaling activation to restrain a robust immune pressure. They would then progressively evolve to conquer the neighboring tissues while their immunophenotype swings toward a more immunosuppressive TAM infiltration pattern with metabolism signaling pathway enrichment in GC tumors. Tumors with enriched TAMs infiltrate would represent advanced stages of GC in full progression and be virtually resistant to the host’s immune system. A number of previous studies have explored the immune microenvironment of gastric cancer through TCGA and GEO databases, showing a similar trend to our results, which validate our CyTOF data well (43, 44). Other studies identified several prognosis-related genes and further constructed a prognostic model of gastric cancer patients based on public databases (45–49). Elucidating the detailed mechanism underlying the tumor-specific immune landscape of GC remains a future challenge to be tackled in the fields of tumor biology and immunology. In our study, the dynamic properties of diverse immune cell types revealed by combined CyTOF, immunohistochemistry, and bioinformatic analysis not only add new dimensions to the immune landscape of GC but also guide the appropriate combinational immunotherapy dependent on the immune signature of GC patients.

It should be noted that our study has limitations. Cell phenotype correlations and associations with tumor progression are based on a cohort of limited few GC patients. Larger and independent cohorts need to be analyzed to yield statistical power sufficient to identify relationships between additional immune phenotypes and clinical outcome of GC. Moreover, due to the limited number of channels of CyTOF, it is hard for us to cover all of immune-related markers for more specific subsets such as functional analysis of T cells, B cells, and more immune checkpoints. Even with a limited number of cases, we still found some obvious trends of the change and we have further proved the results using bioinformatic analysis as well. Based on the above, the results remain clinically useful and extensive. We hope, in the future, we can confirm our study by further enlarging the sample size and extending it to more subgroup analyses such as the comparison between intestinal and diffuse form, as well as a deeper analysis of various functional phenotypes such as the exhausted T-cell phenotype.

In summary, we conducted an in-depth and comprehensive analysis of gastric cancer TIME. By drawing an immune panoramic map, we analyzed the composition and functional status of immune cells in gastric cancer TIME, thus showing the connection between immune cells and their relations to tumor progression. This study is of great value for the discovery of early diagnosis, efficacy prediction, and prognosis evaluation of gastric cancer, providing a basis for the discovery of synergistic gastric cancer immunotherapy and new therapeutic targets for gastric cancer.

## Materials and Methods

### Collection of Human Samples and Clinical Characteristics

Cancer tissues, adjacent tissues, and blood were obtained from GC patients with radical resection (all patients did not undergo radiotherapy, chemotherapy, and immunotherapy before surgery) at the National Cancer Center/Cancer Hospital, Chinese Academy of Medical Sciences, and Peking Union Medical College or Beijing Friendship hospital, Capital Medical University from January 2018 to January 2019.

A total of 49 cases were collected, among which 39 cases were rejected due to disqualification. Finally, 10 patients were eligible for sample collection of paired gastric cancer tissues and adjacent tissue tissues, among which eight had the matched peripheral blood samples. Baseline information such as clinical data of patients (history of helicobacter pylori infection, smoking history, drinking history, family history of cancer, accompanied diseases, etc.) and pathological data (type, grade, stage, pathological classification, degree of differentiation, the expression of c-MET, EGFR, HER2, etc.) was collected at the same time.

Preoperative peripheral blood was collected within 4 h before surgery. Cancer and adjacent tissues were obtained during surgical removal. Cancer tissues with good vitality were cut out, and adjacent tissues were taken 2 centimeters (cm) away from cancer. The specimen was 1 cm in diameter and weighed 100–200 milligrams (mg). After resection, tissues were reserved in RPMI 1640 on ice and transported to the laboratory for immediate processing.

### Tissue Digestion and PBMC Isolation

Samples were respectively prepared for single-cell suspension within 2 h for mass cytometry. The samples were washed with sterile saline and mechanically digested into 1-mm3 fragments; homogenized twice with gentleMACS Dissociator B-01 mode in HBSS containing 0.03% type IV collagenase, 0.01% DNase I, and 10% fetal bovine serum and then digested at 37°C for 45 min. Then, 10 ml MACS was added to neutralize the digestive enzymes. Tissues were filtered through 50 mesh and 70 mesh filters, respectively, rinsed with MACS, and centrifuged at 300g for 4 min to collect the sediment; MACS suspension cells were single cells of gastric cancer/adjacent tissues.

The PBMCs were collected via Ficoll. PBMCs were washed and resuspended in cell staining buffer (CSB) to create a single-cell suspension. Single-cell samples were suspended in phosphate-buffered saline (PBS) and stained with 0.5 μM cisplatin at room temperature. CSB was added to stop staining. Samples were then centrifuged at 500g for 5 min at room temperature. Cells were resuspended, and 3.2% PFA was added to fix the samples. DMSO was applied for cryopreservation.

### Antibody Staining and Data Acquisition for CyTOF

Most of the antibodies in the study were commercially pre-conjugated. Others were conjugated using Maxpar Multimetal Labeling Kits according to the manufacturer’s protocol. Briefly, purified antibodies were exchanged with a buffer using 50-kDa ultrafiltration columns and partially reduced with 4 mM TCEP (Thermo Scientific, Waltham, MA, USA) and then conjugated to their respective lanthanide-loaded polymers and washed. The final conjugated antibodies were diluted to a final concentration of 0.5 mg/ml in the antibody stabilization buffer containing 0.05% sodium azide and stored at 4°C before staining.

Frozen cells were washed and recovered with CSB. An extracellular antibody cocktail was added to stain the antigens on the cell surface. After the CSB washing, nuclear antigen staining buffer was used to break the cell membrane. Washed by nuclear antigen staining perm, an intracellular antibody cocktail was applied to stain intracellular antigens. After PBS washing, cells were resuspended in cell intercalation solution to incubate overnight. Incubated samples were washed with CSB and ddH2O and then prepared with 10% EQ beads for loading.

Mass cytometry data were finally acquired on the Helios™ mass cytometer (Fluidigm) in the Beijing Institute of Hepatology. Experimental consumables and reagents were summarized in **Supplementary Table S1**.

### Clustering and Dimension Reduction

Randomization, bead normalization, and bead removal of data collection were performed on CyTOF software (Fluidigm) v6.7. Files (.fcs) were uploaded into Cytobank (https://www.cytobank.org/), and populations of interest were gated. Debarcoding was carried out by manual gating as well. Events of interest were CD45+ cells, and each individual sample was exported as a separate.fcs file for the subsequent analysis.

R 3.6.1 (https://www.R-project.org) was applied for further data analysis. CyTOF data were visualized using viSNE, a dimensionality reduction method that uses the Barnes–Hut acceleration of the t-distributed stochastic neighbor embedding (t-SNE) algorithm (50). The Phenograph algorithm was applied for cluster analysis by the expression patterns of single cells (51). To identify the major immune lineages, manual reorganization based on the cluster was conducted.

### CyTOF Statistical Analysis

T cells, B cells, NK cells, myeloid cells, and their subgroups were classified to analyze the internal relationship between immune cells. Paired sample t-test was applied to describe the differences among cancer, adjacent tissue, and PBMCs of the same patient. The relationship between immune cells and clinicopathological characteristics was performed using a t-test. Values in the figures are shown as mean ± SEM, with P < 0.05 as the significant difference standard. Statistical calculations were performed using GraphPad Prism 8.0.2 (GraphPad Software Inc., La Jolla, CA, USA).

### Immunohistochemistry

Tissue sections were dewaxed and hydrated, and antigens were repaired with endogenous catalase blocked. Diluted antibodies (anti-CD3, anti-CD4, anti-CD8) were added in the wet box at 4°C overnight, followed by addition of biotin-conjugated secondary antibodies. After laying in wet box at 37°C for 20 min, streptavidin-horseradish peroxidase was added to show colors. Counterstaining, dehydration, transparency, and sealing were done afterward.

### Bioinformatic Analysis

The transcriptome expression profiles and corresponding clinical information of gastric adenocarcinomas were downloaded from the Genomic Data Commons Data Portal of TCGA (https://cancergenome.nih.gov/) and GEO. The Cibersort algorithm was used to analyze the immune cell components of all samples (52), resulting in 22 immune cell subtypes being defined. Only cases with a Cibersort p < 0.05 were included in the subsequent analysis. The differential infiltrations of the 22 immune cell types were evaluated by the Wilcoxon test. Correlations among different immune cells were tested by the corrplot R package. Wilcoxon test was performed to analyze correlations between filtered immune cells and stage.

Upregulated pathways were identified among tumors of the two immunophenotypes by gene set enrichment analysis (GSEA) (pathways for which |NES| >1 and NOM p < 0.05 were chosen to be shown). The gene sets selected for enrichment analysis included GO (BP, MF), KEGG, Hallmark, and Reactome. TIMER (Tumor Immune Estimation Resource) was used to conduct Kaplan–Meier analysis for survival grouped by immune cells.

All bioinformatics analyses were conducted using R 3.6.1 (https://www.R-project.org), GSEA software (http://software.broadinstitute.org/gsea/doc/GSEAUserGuideFrame.html? Interpreting GSEA), and TIMER2.0 (https://cistrome.shinyapps.io/timer/).

### Study Approval

The study was approved by the Ethics Committee of Beijing Friendship Hospital, Capital Medical University. All patients had signed informed consent for sample collection and clinical data collection.

## Data Availability Statement

The original contributions presented in the study are included in the article/**Supplementary Material**. Further inquiries can be directed to the corresponding authors.

## Ethics Statement

The studies involving human participants were reviewed and approved by 2017-P2-141-01. The patients/participants provided their written informed consent to participate in this study.

## Author Contributions

Conception and design: QL, XY, HL, and YX. Development of methodology: XF, YW, XJ, LY, MW, MH, and YRL. Acquisition of data (acquired and managed patients, provided facilities, etc.): YW, JZ, DC, and ZZ. Analysis and interpretation of data (e.g., statistical analysis, biostatistics, computational analysis): YW, YTL, XY, QD, and DW. Writing, review, and/or revision of the manuscript: XY, YW, XJ, YFL, and QL. Administrative, technical, or material support (i.e., reporting or organizing data, constructing databases): JJ, QL, and YW. Study supervision: QL, XY, and YW. All authors contributed to the article and approved the submitted version.

## Funding

This work was supported by the National Natural Science Foundation of China (81301912, 81772525), the Beijing Municipal Health and Scientific and Technological Achievements and Appropriate Technology Promotion Project (BHTPP202008), the “High-value Patent Cultivation” project of the Beijing Friendship Hospital Affiliated to the Capital Medical University (yyzscq202003), and the Research Foundation of Beijing Friendship Hospital (yyqdkt2019-40).

## Conflict of Interest

Author DW is employed by CNNC Hexin Information Technology Beijing Co., Ltd.

The remaining authors declare that the research was conducted in the absence of any commercial or financial relationships that could be construed as a potential conflict of interest.

## Publisher’s Note

All claims expressed in this article are solely those of the authors and do not necessarily represent those of their affiliated organizations, or those of the publisher, the editors and the reviewers. Any product that may be evaluated in this article, or claim that may be made by its manufacturer, is not guaranteed or endorsed by the publisher.
