# Supplementary material for: Immune Profiling in Gastric Cancer Reveals the Dynamic Landscape of Immune Signature Underlying Tumor Progression
Source: Front Immunol. 2022 Jul 8;13:935552. doi: 10.3389/fimmu.2022.935552 (PMC9304688; doi:10.3389/fimmu.2022.935552)
Supplement: Supplementary file 1 [file DataSheet_1.pdf]

Supplementary Material

Immune Profiling in Gastric Cancer Reveals the Dynamic Landscape of Immune Signature Underlying Tumor Progression

Yuhan Wei[1]*, Jianwei Zhang[2]*, Xueke Fan[3]*, Zhi Zheng[4]*, Xiaoyue Jiang[1]*, Dexi Chen[5]*, Yuting Lu[1], Miao Wang[1], Min Hu[6,1], Yingrui Li[1], Qi Du[1], Liuting Yang[6], Hongzhong Li[7], Yi Xiao[8], Yongfu Li[1,9], Jiangtao Jin[10], Deying Wang[11], Xiangliang Yuan[12#], Qin Li[1#]

[#]Corresponding Author:
Qin Li; E-mail: qinli128003@ccmu.edu.cn
Xiangliang Yuan; E-mail: yuanxiangliang@gmail.com

This supplementary file includes:

Supplementary Fig. S1 to S5

Supplementary Table S1, S2

Supplementary Figures

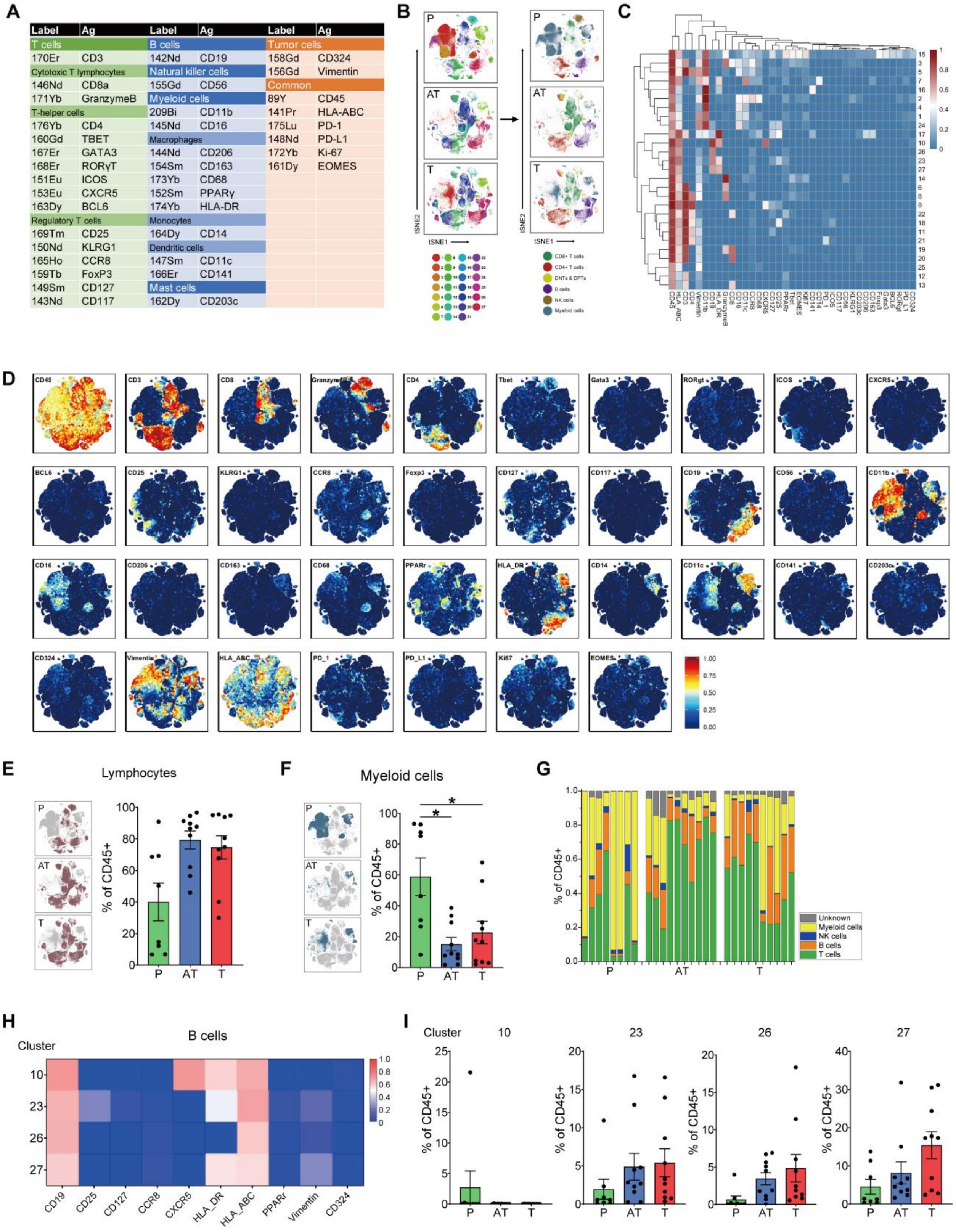

Fig S1. Mass Cytometry Analysis Reveals Unique Changes in the Composition of $CD45^+$ Immune Cells in Human Gastric Cancer, Related to Fig 1.

(A) Markers used to characterize immune phenotypes.

(B) tSNE maps displaying immune cells of GC patients in the three types of samples (peripheral blood mononuclear cells [PBMCs, P], adjacent tissues [AT] and tumor tissues [T]) colored by 27 Phenograph clusters (left) and the main cell populations by manual identification of Phenograph clustering (right).

(C) Heatmap displaying normalized expression of the pre-designed 37 markers for the 27 Phenograph clusters.

(D) tSNE maps displaying the relative expression of the 37 CyTOF markers for all samples.

(E, F) Distribution of lymphocytes and myeloid cells in different types of GC samples and frequency of that for each patient based on manual identification of Phenograph clusters.

(G) Stacked histogram of the immune composition for the individual by the three types of samples.

(H) Heatmap displaying a normalized expression of the discrepant markers for the 4 Phenograph clusters of B cells.

(I) Frequency of B cell clusters for each patient based on summation of Phenograph clusters.

Bar plots show mean ± SEM; *p < 0.05 by paired t-test. P: PBMCs; AT: adjacent tissues; T: tumor tissues.

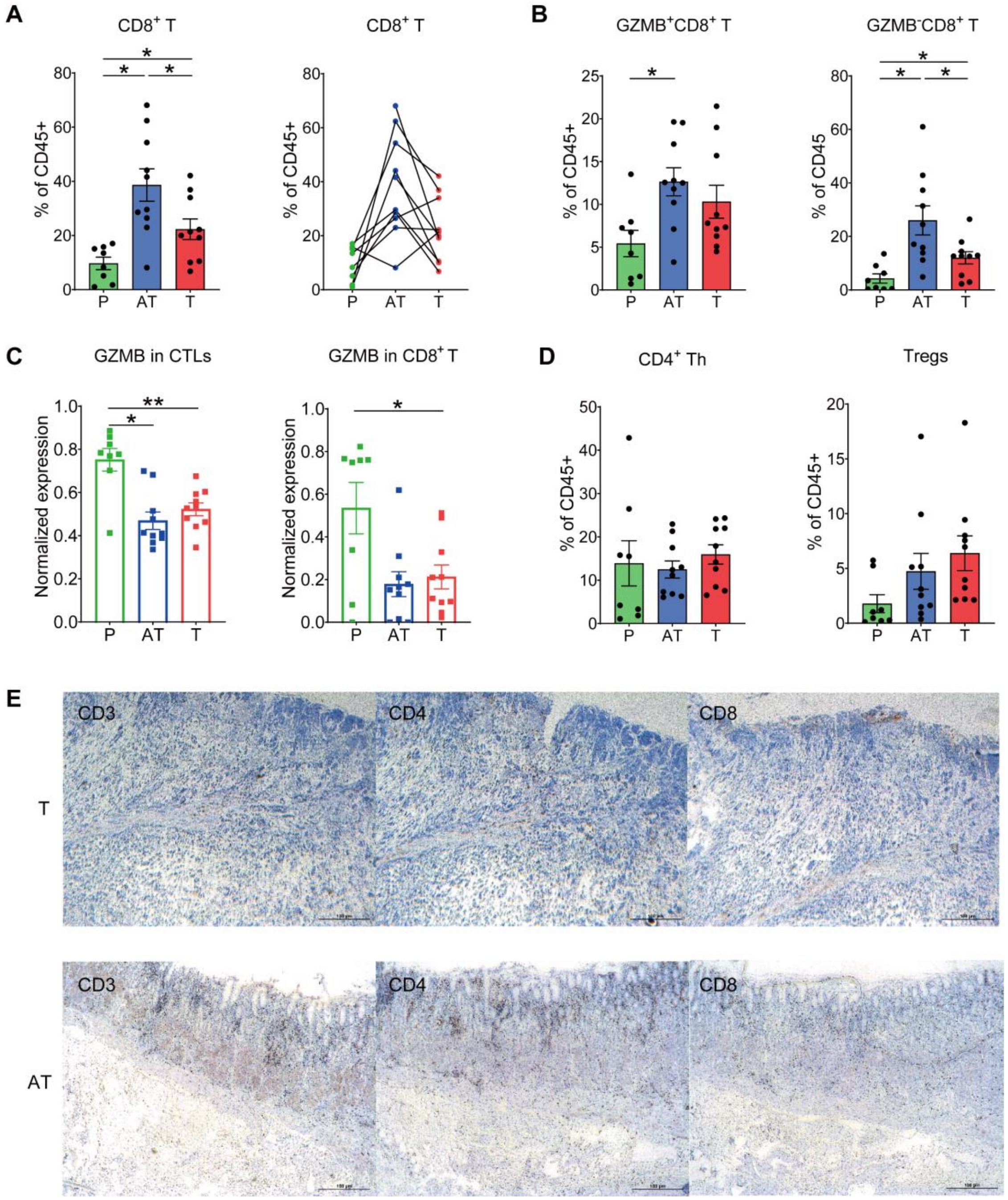

Fig S2. In-Depth Characterization of the Amount and Function of T Cell Compartments, Related to Fig 2.

(A) Frequency of $CD8^+$ T cell clusters for each patient based on summation of Phenograph clusters (% of $CD45^+$ Immune Cells).

(B) Frequency of $GZMB^+CD8^+$ (left) and $GZMB^-CD8^+$ (right) T cell clusters for each patient based on summation of Phenograph clusters (% of $CD45^+$ Immune Cells).

(C) Expression level of GZMB in $GZMB^+CD8^+$ and $CD8^+$ T cells.

(D) Frequency of $CD4^+$ Th and Treg cells for each patient based on summation of Phenograph clusters (% of $CD45^+$ Immune Cells).

(E) The immunohistochemical expressions of CD3, CD4, and CD8 in tumor tissues and adjacent tumor tissues.

Bar plots show mean ± SEM; *$p < 0.05$, **$p < 0.01$ by paired t-test.

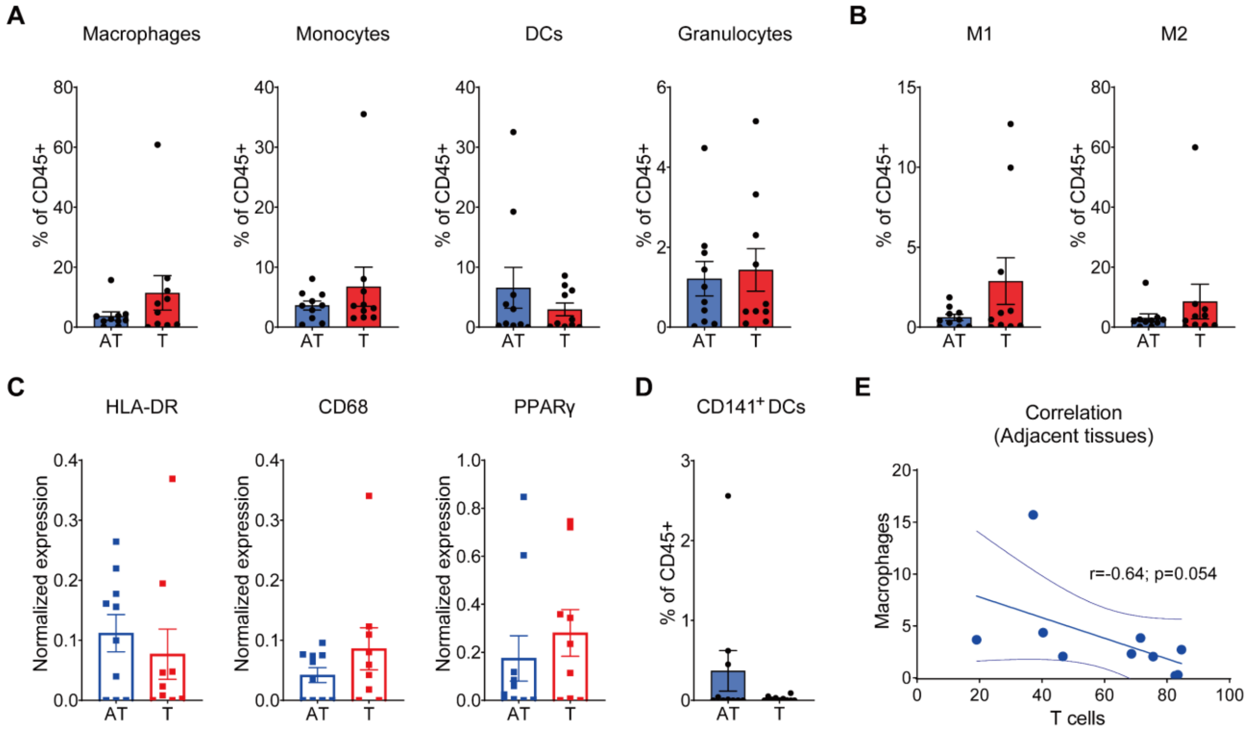

Fig S3. Myeloid Cells Show a Distinct Phenotype in GC Immune Microenvironment (% of $CD45^+$ Immune Cells), Related to Fig 3

(A) Frequency of macrophages, monocytes, DCs and granulocytes for each patient based on summation of Phenograph clusters.

(B) Frequency of M1-type and M2-type macrophages for each patient based on summation of Phenograph clusters.

(C) Expression level of HLA-DR, CD68, and PPARγ in macrophages.

(D) Frequency of $CD141^+$ DCs for each patient based on summation of Phenograph clusters.

(E) Correlation between T cells and macrophages in all adjacent tissues. Spearman's correlation r and p values are shown.

Bar plots show mean ± SEM. DCs: dendric cells; M1: M1-type macrophages; M2: M2-type macrophages.

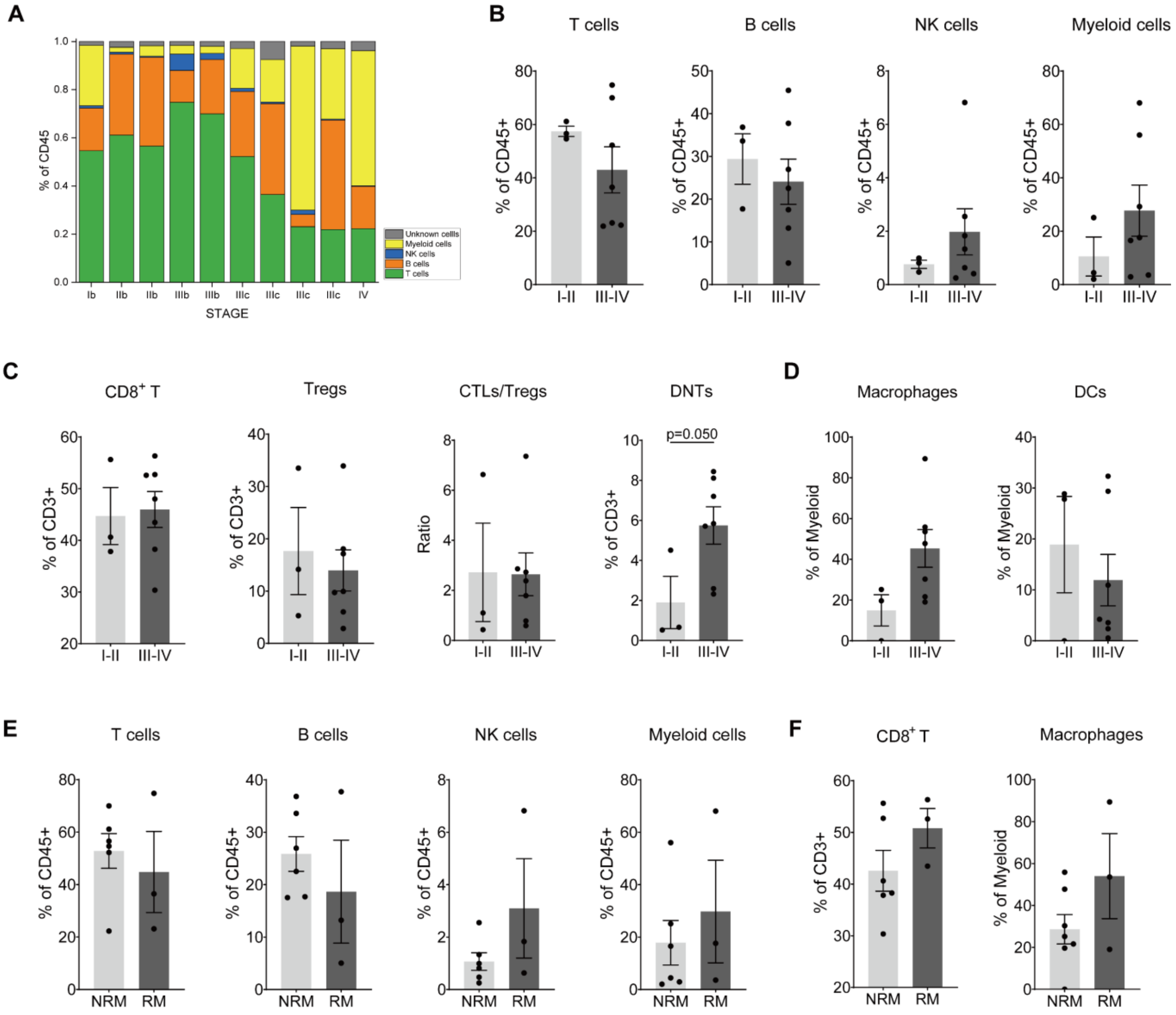

Fig S4. Dynamic Signature of the Immune Landscape Underlying GC Tumor Progression, Related to Fig 4

(A) Stacked histogram of the immune composition of individual by stage.

(B) Frequency of T, B, NK, and myeloid cells in tumor tissue for each patient by stage (% of $CD45^+$ immune cells).

(C) Frequency of $CD8^+$ T cells, Tregs, the ratio of CTL/Treg (left), and frequency of DNT cells (% of $CD3^+$ immune cells) for each patient by stage.

(D) Frequency of macrophages and DCs for each patient by stage (% of myeloid cells).

(E) Frequency of T, B, NK, and myeloid cells for each patient by RM state (% of $CD45^+$ immune cells).

(F) Frequency of $CD8^+$ T cells and macrophages for each patient by RM state (% of $CD3^+$ and myeloid cells).

Bar plots show mean ± SEM. RM: recurrence and metastasis; NRM: no recurrence and metastasis.

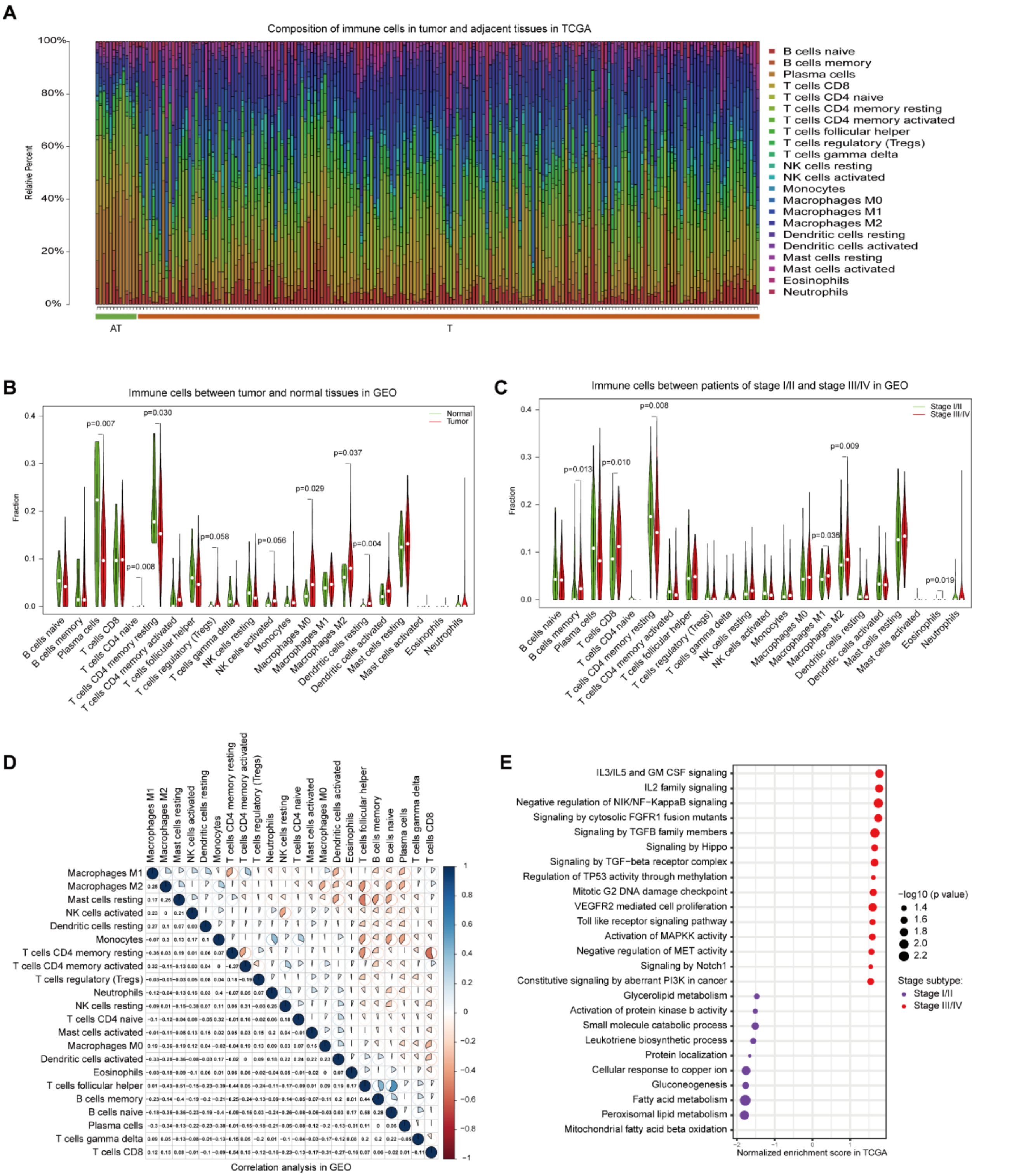

Fig S5. Tumor Immunophenotypes and Relevant Pathways in TCGA and GEO, Related to Fig 5

(A) Fractions of the 22 immune cells defined by CIBERSORT in GC tumor and adjacent normal samples in TCGA.

(B) Comparisons of immune cells between tumor and normal tissues of GC patients in GEO.

(C) Comparisons of immune cells between the GC patients with stage I/II and stage III/IV in GEO.

(D) Correlation of various immune cells defined by CIBERSORT in GEO..

(E) Pathways related to the stage (I/II vs III/IV) of GC patients by GSEA. Pathways for which the $|NES|>1$, $p<0.05$ are chosen to be shown. The position of each circle represents the normalized enrichment score of immunophenotype in which the upregulated pathway is detected in GC patients. The size of the circles represents -log10 (*P*-value).

GSEA: Gene Set Enrichment Analysis; NES: Normalized Enrichment Score.

Supplementary Tables

Table S1. Experimental Consumables and Reagents

Company	Identifier	Description
Candor Biosciences	131050	Antibody Stabilizer based on PBS 50ml
Millipore	UFC500396	Amicon Ultra-0.5 Centrifugal Filter Unit with Ultracel-3 membrane
Millipore	UFC505096	Amicon Ultra-0.5 Centrifugal Filter Unit with Ultracel-50 membrane
Pierce	77720	Bond-Breaker™ TCEP Solution, Neutral pH 5ml
Fluidigm	201300	Maxpar® X8 Multimetal Labeling Kit—40 Rxn
Fluidigm	201192A	Cell-ID™ Intercalator-Ir—125 µM
Fluidigm	201064	Cell-ID™ Cisplatin—100 µL
Fluidigm	201068	Maxpar® Cell Staining Buffer—500 mL
Fluidigm	201078	EQ™ Four Element Calibration Beads—100 mL
Fluidigm	201067	Maxpar® Fix and Perm Buffer—100 mL
Fluidigm	201063	Maxpar® Nuclear Antigen Staining Buffer Set—120 Tests
Fluidigm	3151020B	Anti-CD278/ICOS (C398.4A)-151Eu—100 Tests
Fluidigm	3143001B	Anti-Human CD117/c-kit (104D2)-143Nd—100 Tests
Fluidigm	3209003B	Anti-Human CD11b/Mac-1 (ICRF44)-209Bi—100 Tests
Fluidigm	3147008B	Anti-Human CD11c (Bu15)-147Sm—100 Tests
Fluidigm	3149011B	Anti-Human CD127/IL-7Ra (A019D5)-149Sm—100 Tests
Fluidigm	3166017B	Anti-Human CD141 (M80)-166Er —100 Tests
Fluidigm	3145008B	Anti-Human CD16 (3G8)-145Nd—100 Tests
Fluidigm	3154007B	Anti-Human CD163 (GHI/61)-154Sm—100 Tests
Fluidigm	3153020B	Anti-Human CD185/CXCR5 (RF8B2)-153Eu—100 Tests
Fluidigm	3142001B	Anti-Human CD19 (HIB19)-142Nd—100 Tests
Fluidigm	3169003B	Anti-Human CD25 (2A3)-169Tm—100 Tests
Fluidigm	3148017B	Anti-Human CD274/PD-L1 (29E.2A3)-148Nd—100 Tests
Fluidigm	3175008B	Anti-Human CD279/PD-1 (EH12.2H7)-175Lu—100 Tests
Fluidigm	3170001B	Anti-Human CD3 (UCHT1)-170Er —100 Tests
Fluidigm	3176010B	Anti-Human CD4 (RPA-T4)-176Yb—100 Tests
Fluidigm	3089003B	Anti-Human CD45 (HI30)-Y89—100 Tests
Fluidigm	3155008B	Anti-Human CD56 (B159)-155Gd—100 Tests
Fluidigm	3146001B	Anti-Human CD8 (RPA-T8)-146Nd—100 Tests
Fluidigm	3159028A	Anti-Human FoxP3 (259D/C7)-159Tb—50 Tests
Fluidigm	3171002B	Anti-Human Granzyme B (GB11)-171Yb —100 Tests
Fluidigm	3141010B	Anti-Human HLA-ABC (W6-32)-141Pr—100 Tests
Fluidigm	3174001B	Anti-Human HLA-DR (L243)-174Yb—100 Tests
Fluidigm	3172024B	Anti-Human Ki-67 (B56)-172Yb—100 Tests
Fluidigm	3163012B	Anti-Human/Mouse BCL-6 (K112-91)-163Dy—100 Tests
Fluidigm	3158018B	Anti-Human/Mouse CD324/E-Cadherin (DECMA-1)-158Gd—100 Tests
Fluidigm	3167007A	Anti-Human/Mouse Gata3 (TWAJ)-167Er—50 Tests
Fluidigm	3160010B	Anti-Human/Mouse Tbet (4B10)-160Gd—100 Tests

Fluidigm	3156023A	Anti-Vimentin (RV202)-156Gd—50 Tests

**Company**	**Identifier**	**Description**
BioLegend	324602	Purified anti-human CD203c (E-NPP3)
BD Pharmingen	556059	HU CD68 PURE MAB 0.1MG Y1/82A
BioLegend	321127	Purified anti-human CD206 (MMR)
BD Pharmingen	555396	HU CD14 PURE MAB 0.1MG M5E2
BioLegend	360602	Purified anti-human CD198 (CCR8)
BioLegend	368602	Purified anti-human KLRG1 (MAFA)
eBioscience	14-4877-82	Anti-human EOMES purified 100µg
eBioscience	14-6988-82	Purified Anti-mouse/human RORg 100µg
BioLegend	683402	Purified anti-PPAR-γ 100µg

**Company**	**Identifier**	**Description**
HyClone	SH30809.01B	RPMI 1640 HYCLONE
GE Healthcare	17-0891-02	Percoll
Corning cellgro	21-031-CVR	Dulbecco’s Phosphate-Buffered Saline
Sigma	A7030-10G	Bovine serum albumin
Sigma	EDS-100G	Ethylenediaminetetraacetic acid
Sigma	C5138	Collagenase Ⅳ
Sigma	D5025	DNase I
Sigma	P6148	Paraformaldehyde
Sigma	D5879	Dimethyl sulfoxide

**Company**	**Identifier**	**Clone**	**Titer**	**Description**
Maxim	MAB-0740	MX036	1:50	Anti-Human CD3（Immunohistochemistry）
Maxim	RMA-0620	SP35	1:100	Anti-Human CD4 (Immunohistochemistry)
Maxim	MAB-0021	C8/144B	1:50	Anti-Human CD8（Immunohistochemistry）

Table S2. The Clinicopathological Characteristics of GC Patients

Clinical Characteristic	N (%)	Clinical Characteristic	N (%)
**Age (years)**		**Tumor differentiation**	
<65	8 (80)	Moderate differentiation	2 (20)
⩾65	2 (20)	Poor differentiation	8 (80)
**Gender**		**EBV**	
Male	8 (80)	+	0 (0)
Female	2 (20)	-	2 (20)
**T stage**		Unknown	8 (80)
T1-T3	3 (30)	**HER2 status**	
T4	7 (70)	+	1 (10)
**N stage**		-	9 (90)
N0-N2	5 (50)	**c-MET status**	
N3	5 (50)	+	4 (40)
**M stage**		-	3 (30)
M0	9 (90)	Unknown	3 (30)
M1	1 (10)	**EGFR status**	
**Stage**		+	5 (50)
I+II	3 (30)	-	2 (20)
III+IV	7 (70)	Unknown	3 (30)
**Tumor site**		**MSH2 status**	
Gastric body	1 (10)	+	7 (70)
Antrum	2 (20)	Unknown	3 (30)
Pylorus	2 (20)	**MSH6 status**	
Mixed	5 (50)	+	7 (70)
**Pathological type**		Unknown	3 (30)
Adenocarcinoma	7 (70)	**PMS2 status**	
Adenocarcinoma with SRC	3 (30)	+	7 (70)
**Lauren type**		Unknown	3 (30)
Diffuse	6 (60)	**MLH1 status**	
Intestinal	3 (30)	+	7 (70)
Mixed	1 (10)	Unknown	3 (30)

Abbreviations: GC: gastric cancer; T: tumor; N: lymph node; M: metastasis; HER-2: human epidermal growth factor receptor 2; EGFR: epidermal growth factor receptor; SRC: signet ring cell.
